# Phase Diagrams of n-Type Low Bandgap Naphthalenediimide-Bithiophene Copolymer Solutions and Blends

**DOI:** 10.3390/polym11091474

**Published:** 2019-09-09

**Authors:** Gada Muleta Fanta, Pawel Jarka, Urszula Szeluga, Tomasz Tański, Jung Yong Kim

**Affiliations:** 1 Institute of Engineering Materials and Biomaterials, Faculty of Mechanical Engineering, Silesian University of Technology, 44-100 Gliwice, Poland; 2 School of Materials Science and Engineering, Jimma Institute of Technology, Jimma University, Post Office Box 378 Jimma, Ethiopia; 3 Center of Polymer and Carbon Materials to the Polish Academy of Sciences, M. Curie-Skłodowska 34 Street, 41-819 Zabrze, Poland; 4 School of Chemical Engineering, Jimma Institute of Technology, Jimma University, Post Office Box 378 Jimma, Ethiopia

**Keywords:** phase diagram, Flory–Huggins theory, n-type polymer, low bandgap polymer, conjugated polymer, polymer solution, polymer blend, all polymer solar cells

## Abstract

Phase diagrams of n-type low bandgap poly{(*N*,*N*′-bis(2-octyldodecyl)naphthalene -1,4,5,8-bis(dicarboximide)-2,6-diyl)-alt-5,5′,-(2,2′-bithiophene)} (P(NDI2OD-T2)) solutions and blends were constructed. To this end, we employed the Flory–Huggins (FH) lattice theory for qualitatively understanding the phase behavior of P(NDI2OD-T2) solutions as a function of solvent, chlorobenzene, chloroform, and p-xylene. Herein, the polymer–solvent interaction parameter (χ) was obtained from a water contact angle measurement, leading to the solubility parameter. The phase behavior of these P(NDI2OD-T2) solutions showed both liquid–liquid (L–L) and liquid–solid (L–S) phase transitions. However, depending on the solvent, the relative position of the liquid–liquid phase equilibria (LLE) and solid–liquid phase equilibria (SLE) (i.e., two-phase co-existence curves) could be changed drastically, i.e., LLE > SLE, LLE ≈ SLE, and SLE > LLE. Finally, we studied the phase behavior of the polymer–polymer mixture composed of P(NDI2OD-T2) and regioregular poly(3-hexylthiophene-2,5-dyil) (r-reg P3HT), in which the melting transition curve was compared with the theory of melting point depression combined with the FH model. The FH theory describes excellently the melting temperature of the r-reg P3HT/P(NDI2OD-T2) mixture when the entropic contribution to the polymer–polymer interaction parameter (χ = 116.8 K/*T* − 0.185, dimensionless) was properly accounted for, indicating an increase of entropy by forming a new contact between two different polymer segments. Understanding the phase behavior of the polymer solutions and blends affecting morphologies plays an integral role towards developing polymer optoelectronic devices.

## 1. Introduction

The phase behavior of conjugated polymer–solvent and polymer–polymer mixtures has been an interesting topic of research in which there are two important phase-separation mechanisms, i.e., liquid–liquid (L–L) and liquid–solid (L–S) phase transitions [1,2,3]. Herein, the L–L phase transition is similarly divided into two: spinodal decomposition (SD), and nucleation and growth (NG) [4,5,6]. The former proceeds in an unstable region through spontaneous phase separation without energy barriers, leading to a high interconnectivity of two phases, whereas the latter exists in a metastable region [4,7,8]. Specifically, in the field of polymer optoelectronics, L–L or amorphous–amorphous phase transition through SD has been emphasized because the bicontinuous phase morphologies of polymer/fullerene or polymer/polymer resemble those generated through SD demixing [6,9]. However, many well-known conjugated polymers, including regioregular poly(3-hexylthiophene -2,5-diyl) (r-reg P3HT) and naphthalenediimide-bithiophene copolymer (P(NDI2OD-T2)), are semicrystalline, not pure amorphous, indicating that they may exhibit not only liquid–liquid phase equilibria (LLE) but also solid–liquid phase equilibria (SLE), i.e., self-assembly for crystallization [10,11] in the thin-film process from solution [2,3]. Hence, in the case of stereoregular polymer-based solutions, it becomes very important to elucidate both SLE and LLE mechanisms and their sequences (SLE to LLE or LLE to SLE) for understating morphology-formation mechanisms. 

Recently, all polymer solar cells (all-PSCs) with active layers composed of polymer donor (P_D_) and polymer acceptor (P_A_) have been competitive technologies as an alternative to polymer–fullerene solar cells (PFSCs) [12,13,14,15,16]. Increasing the efficiency of photovoltaic devices based on the use of organic materials, especially in the form of nanostructure elements, is the object of the research of scientists from around the world [17,18,19,20,21,22,23,24]. Currently, state-of-the-art single-junction all-PSCs show a power conversion efficiency (PCE) over 10%, which still lags behind PFSCs, demonstrating a PCE of over 16% [25,26]. However, all-PSCs have clear advantages compared to PFSCs in that they have electronic tunability, leading to high open-circuit voltage and light absorption, thermodynamic stability in morphologies, mechanical stability of devices, lower cost of synthesis, large-scale processability in manufacturing, and others. 

In 2009, Facchetti et al. reported that P(NDI2OD-T2) has a high electron mobility of ~0.85 cm^2^/Vs [27]. Since then, P(NDI2OD-T2) has been mostly used as a benchmark P_A_ in all-PSCs, based on its properties, including high electronic charge mobility, a small bandgap (1.45 eV) leading to effective light absorption, high electron affinity in its lowest unoccupied molecular orbital (LUMO = −4.0 eV), density (1.1 g/cm^3^), glass transition temperature (*T*_g_ ≈ −70 °C), aggregation in common solvents, and controllable face-on or edge-on molecular orientation depending on the molecular weight and its distribution [28,29,30,31,32,33,34,35,36,37]. However, in spite of the aforementioned strong characteristics, all-PSCs based on r-reg P3HT/P(NDI2OD-T2) showed a very low PCE (~0.2%) initially, because of the geminate recombination of charge pairs originating from its coarse phase separation with a large domain size of ~0.2–1 μm [28]. Note that organic semiconductors, π-conjugated polymers, and small molecules have low dielectric constants and van der Waals bonding [38,39]. Hence, for separating small radius (<5 Å) Frenkel excitons, a phase-separation scale around the exciton diffusion length (~10 nm) must be controlled, leading to a sufficient interfacial area [38]. Hence, for effectively controlling morphologies, we need to understand the phase-separation mechanism in detail.

Previous studies [2] showed that the r-reg P3HT solution exhibits L–S phase transition related to order–disorder phase equilibria between single-coiled polymer in solution and polymer in nanocrystalline aggregate. The phase diagrams of low bandgap copolymer, poly(2,6-(4,4-bis (2-ethylhexyl)-4H-cyclopenta(2,1-b;3,4-b′)dithiophene)-alt-4,7-(2,1,3-benzothiadiazole)) (PCPDTBT) solutions as a function of solvent, chain length, polymer species, fullerene size, etc., are constructed theoretically [3]. Then, with this understanding of solution phase behavior for crystalline–amorphous, amorphous–amorphous, and amorphous crystalline mixtures, the phase diagrams of binary PCPDTBT: [6,6]-phenyl C_61_ butyric acid methyl ester (PC_61_BM) and PCPDTBT: [6,6]-phenyl C_71_ butyric acid methyl ester (PC_71_BM) blends are constructed based on the thermal and optical properties of the materials [3]. 

In this study, we report that how n-type low bandgap P(NDI2OD-T2) solution undergoes L–L and L–S phase transition depends on the solvent in comparison with the phase behavior of the r-reg P3HT solutions. These phase behaviors are qualitatively described by the Flory–Huggins (FH) lattice theory [1,40,41], for which the *χ* interaction parameter is estimated from contact angle measurements, leading to a solubility parameter [2,3]. Then, the phase behavior of all semicrystalline polymers, the r-reg P3HT/P(NDI2OD-T2) system, is studied based on both experimental and theoretical analyses. Importantly, for analyzing the melting points of r-reg P3HT/P(NDI2OD-T2), we employ the theory of melting point depression combined with the FH model [1,40,41], and observe excellent agreement between the theoretical and experimental results when we employ a χ parameter with both enthalpic and entropic contributions. Note that, when a polymer contains impurities (e.g., solvents or copolymerized units or other polymers), the melting point is shifted by re-establishing the condition of equilibrium between liquid and crystalline polymer, which could be described by combining the melting point depression theory with the FH model [1]. 

## 2. Experimental and Calculation Methods

### 2.1. Materials

P(NDI2OD-T2) (*M*_n_ = 32.1 kg/mol, *M*_w_ = 90.0 kg/mol, polydispersity index (PDI) = 2.8, and molecular formula = (C_62_H_88_N_2_O_4_S_2_)_n_) was purchased from 1-Material Inc. (Dorval, Quebec). R-reg P3HT (*M*_n_ = 29.6 kg/mol, *M*_w_ = 65.2 kg/mol, PDI = 2.2, and molecular formula = (C_10_H_14_S)_n_) was acquired from Sigma-Aldrich Inc. (Taufkirchen, Germany). All these materials were used as received without further purification. 

### 2.2. Contact Angle Measurement 

In order to determine the wetting ability of surfaces on thin-film samples of P(NDI2OD-T2) and r-reg P3HT, an analysis of contact angles (θ) was carried out. Measurements of the contact angle were made using distilled water. The measurement of a drop of liquid applied to the surfaces of the samples (i.e., a spin-coated film on glass substrate) was made on the OEG SURFTENS UNIVERSAL test bench. Five drops of distilled water, each with a volume of 1 μL, were applied to the surface of each sample. The measurement was taken 15 s from the moment the drop was applied. Then the contact angles were observed and the mean values with standard deviation were calculated. 

### 2.3. Solubility Parameter Calculation 

According to Li and Neumann [42,43], the contact angle (θ) can be expressed as follows:

(1) $$\cos\theta =-1+2\sqrt{\frac{\gamma_{\mathrm{sv}}}{\gamma_{\mathrm{lv}}}}e^{-\beta {\left(\gamma_{\mathrm{lv}}-\gamma_{\mathrm{sv}}\right)}^{2}}$$

where *γ*_lv_, *γ*_sv_, and *γ*_sl_ are surface energies for liquid–vapor, solid–vapor, and solid–liquid, respectively, and the constant β is 0.000115 (m^2^/mJ)^2^. Then δ can be obtained based on $\delta \propto \sqrt{\gamma_{\mathrm{sv}}}$ [3].

### 2.4. Thermal Characterization 

Differential Scanning Calorimetery (DSC) (2920-DSC, TA Instruments, Champaign, IL, USA) was performed to characterize the transition temperature of materials at a scan rate of 10 °C/min from 20 to 350 °C under N_2_ according to the instrumental set-up conditions. Thermogravimetric analysis (TGA) was carried out using a METTLER TOLEDO Thermal Analysis (STARe System) (Warsaw, Poland), in which samples were heated from 50 to 600 °C using a conventional heating ramp with a scan rate of 10 °C/min under N_2_. 

## 3. Results and Discussion

### 3.1. Binary Polymer–Solvent Mixture

**Figure 1:**
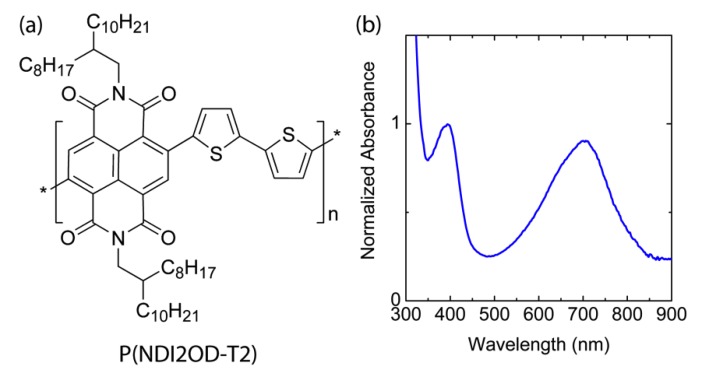
(**a**) Chemical structure of P(NDI2OD-T2). (**b**) UV-VIS spectrum of P(NDI2OD-T2) film.

**Figure 2:**
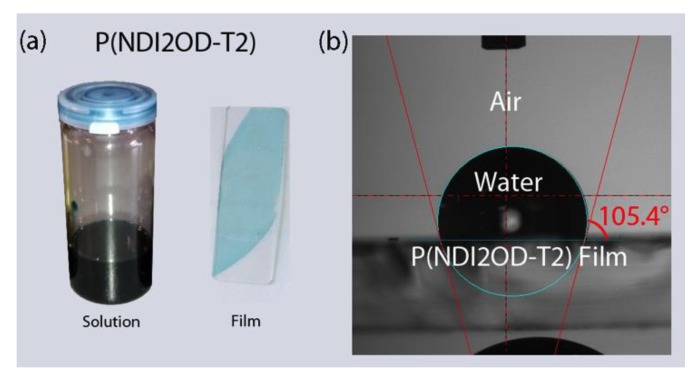
(**a**) P(NDI2OD-T2) solution and film. (**b**) Contact angle measurement for P(NDI2OD-T2) film on glass substrate.

Figure 1 shows the chemical structure of n-type low bandgap P(NDI2OD-T2) and its UV-VIS absorption spectra, harvesting light near infrared regions, about 855 nm. When P(NDI2OD-T2) is dissolved in a common solvent such as chlorobenzene (CB), the solution color is almost black, but its film is bluish, as shown in Figure 2a. For estimating the solubility parameter (δ), we measured the contact angle, as shown in Figure 2b. The calculated solubility parameter and surface energy are summarized in Table 1. As shown in Table 1, P(NDI2OD-T2) has δ = 7.99 (cal/cm^3^)^1/2^ [= 16,386.31 (J/m^3^)^1/2^], whereas r-reg P3HT (*M*_n_ = 29.6 kg/mol, PDI = 2.2) has δ = 9.23 (cal/cm^3^)^1/2^ [= 18,908.58 (J/m^3^)^1/2^], indicating P(NDI2OD-T2) is much more hydrophobic compared to r-reg P3HT. Note that in previous studies [2], when estimating the *δ* of r-reg P3HT (*M*_n_ = 22.0 kg/mol, PDI = 2.1) from the contact angle measurement, a value of 8.72 was obtained. Hence, the average *δ* of r-reg P3HTs with *M*_n_ = ~22.0–29.6 kg/mol could be 8.98 ± 0.36. Table 2 shows some properties, including the *δ* for common solvents [CB, chloroform (CF), and p-xylene (XY)] [44], which are used for studying the phase behavior of n-type P(NDI2OD-T2) solutions in comparison with p-type r-reg P3HT ones. 

According to the FH theory, the molar Gibbs energy of mixing ($\Delta G_{M}$) is given by:

(2) $$\frac{\Delta G_{M}}{RT}=\frac{\phi_{1}}{r_{1}}\ln\phi_{1}+\frac{\phi_{2}}{r_{2}}\ln\phi_{2}+\chi \phi_{1}\phi_{2}$$

where $\phi_{1},\;\phi_{2},\;r_{1}$ (= 1 for solvent in polymer–solvent mixture; ≠ 1 for polymer in polymer–polymer blends), $r_{2}$, *R*, and *T* are the volume fraction, relative molar volumes of component 1 and 2, the gas constant, and temperature (K), respectively. Note that a single lattice site is decided by the molecular volume of the solvent (e.g., CB, CF, and XY) or the polymer′s structural unit for the polymer–polymer blend (e.g., r-reg P3HT′s repeat unit). Herein, χ applies to lattice site volume with a radius of 0.34 nm for CB, 0.31 nm for CF, and 0.36 nm for XY, respectively (Table 2). The FH interaction parameter (χ) can be divided into enthalpic $\left(\chi_{H}\right)$ and entropic $\left(\chi_{S}\right)$ contributions [1,45].

(3) $$\chi =\chi_{H}+\chi_{S}=\frac{z\Delta w_{H}}{kT}-\frac{z\Delta w_{S}}{k}$$

(4) $$\Delta w_{G}=\Delta w_{H}-T\Delta w_{S}$$

where, $\Delta w_{G}$ is the interchange free energy of a segment pair with enthalpic $\left(\Delta w_{H}\right)$ and entropic $\left(\Delta w_{S}\right)$ contributions, *z* is the coordination number (e.g., *z* is in the range of 6 to 12 [1]. Herein, we used *z* = 6 for theoretical calculation), and *k* is the Boltzmann constant. Here, we assign $\chi_{S}$ = 0.34 for the polymer–solvent system. Then, $\chi_{H}$ (dimensionless) can be re-expressed in terms of *δ* as follows:

(5) $$\chi_{H}=\frac{{\hat{V}}_{1}\;}{RT}{\left(\delta_{i}-\delta_{j}\right)}^{2}$$

where ${\hat{V}}_{1}\;\left(=\;\mathrm{lattice}\;\mathrm{site}\;\mathrm{volume}\right)$ and $\delta_{i\;\mathrm{or}\;j}$ are the molar volume of component 1 (solvent) [${\hat{V}}_{1}=\left(112.56\;g/\mathrm{mol}\right)/\left(1.11\;g/{\mathrm{cm}}^{3}\right)$ = 101.41 cm^3^/mol for CB] and the solubility parameter (subscript *i* or *j* = 1, 2; solvent = 1 and polymer = 2 for the polymer–solvent mixture), respectively. Based on Table 1 and Table 2, the estimated $r_{2}\;\mathrm{and}\;\chi_{H}$ values are listed in Table 3. Note that in some experimental observations [46,47], χ is a function of not only a temperature-dependent interaction parameter, D(T), but also a composition-dependent parameter $B\left(\phi_{2}\right)$. Hence, in an extended FH theory, Qian et al. [48] suggested $\chi =D\left(T\right)B\left(\phi_{2}\right)=\left(d_{0}+d_{1}/T+d_{2}\ln T\right)\left(1+b_{1}\phi_{2}+b_{2}\phi_{2}^{2}\right)$, where *d*_0_, *d*_1_, *d*_2_, *b*_1_, and *b*_2_ are adjustable parameters. When χ is independent of concentration, i.e., $B\left(\phi_{2}\right)=1$, χ is recovered to χ=D(T). In this work, when *B*(*ϕ*_2_) = 1, we used $\chi =D\left(T\right)=\chi_{H}+\chi_{S}=d_{0}+d_{1}/T+d_{2}\ln T$, where $d_{0}=0.34=\chi_{S}$, $d_{1}=\left({\hat{V}}_{1}/R\right){\left(\delta_{i}-\delta_{j}\right)}^{2}=\chi_{H}\cdot T$, and $d_{2}=0$ for the polymer–solvent system according to Equations (3) and (5).

**Figure 3:**
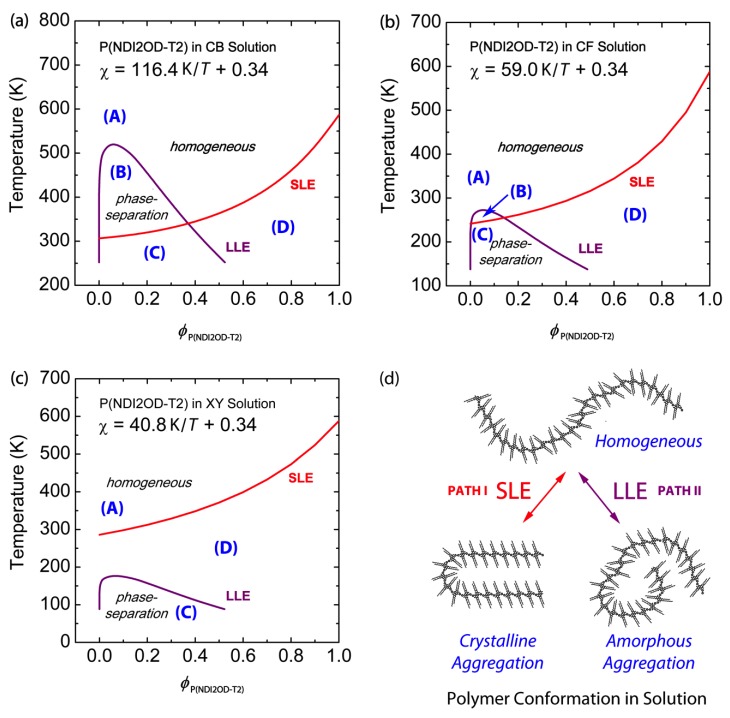
Phase diagrams of binary P(NDI2OD-T2) solutions: Solvent effect. Theoretical phase diagrams of (**a**) P(NDI2OD-T2)/CB, (**b**) P(NDI2OD-T2)/CF, and (**c**) P(NDI2OD-T2)/XY solutions, based on the Flory–Huggins lattice theory. (**d**) Schematic explanation of liquid-liquid phase equilibria (LLE) and solid-liquid phase equilibria (SLE) phase transition of P(NDI2OD-T2) molecules in solution. Herein, Path I indicates SLE (i.e., crystallization), whereas Path II denotes LLE (amorphous–amorphous phase separation). Regions correspond to: (**A**) one-phase liquid state; (**B**) two-phase liquid state; (**C**) both L–L and L–S phase separation; and (**D**) L–S phase separation (i.e., polymer crystallization), respectively.

Figure 3 shows the phase diagram of binary P(NDI2OD-T2)/CB, P(NDI2OD-T2)/CF and P(NDI2OD-T2)/XY solutions, for which Equations (6) and (7) are solved simultaneously:

(6) $$\Delta \mu_{1}^{\alpha }=\Delta \mu_{1}^{\beta }$$

(7) $$\Delta \mu_{2}^{\alpha }=\Delta \mu_{2}^{\beta }$$

where $\Delta \mu_{1}=\partial \Delta G_{M}/\partial n_{1}\;\mathrm{and}\;\Delta \mu_{2}=\partial \Delta G_{M}/\partial n_{2}$ are the chemical potentials of component 1 and 2, respectively, and *α* and *β* indicate two different phases at equilibrium. Then, for describing the melting point depression of the polymer–solvent system, the below equations were used [1,2,3]:

(8) $$\frac{1}{T_{m,2}}-\frac{1}{T_{m,2}^{0}}=-\frac{R}{\Delta H_{u,2}}\frac{V_{u}}{r_{2}{\hat{V}}_{1}}\left[\ln\phi_{2}+\left(1-\frac{r_{2}}{r_{1}}\right)\phi_{1}+r_{2}\chi \phi_{1}^{2}\right]$$

(9) $$\frac{1}{T_{m,2}}-\frac{1}{T_{m,2}^{0}}\approx \frac{R}{\Delta H_{u,2}}\frac{V_{u,2}}{{\hat{V}}_{1}}\left(\phi_{1}-\chi \phi_{1}^{2}\right)\;\left(r_{2}\gg r_{1}\approx 1\right)$$

where $T_{m,2}$ and $T_{m,2}^{0}\left(=587.95\;K\right)$ are the melting temperature of P(NDI2OD-T2) with solvent and the melting temperature of pure P(NDI2OD-T2) without solvent, respectively. Note that in Equation (8), $V_{u}/r_{2}{\hat{V}}_{1}$ is introduced for calculating per structural unit of polymer. $\Delta H_{u,2}$ and $V_{u,2}$ [= (989 g/mol)/(1.1 g/cm^3^) = 899.09 cm^3^/mol] are the unit enthalpy and the unit volume of P(NDI2OD-T2), respectively. Herein, the density of P(NDI2OD-T2) is ca. 1.1 g/cm^3^. However, P(NDI2OD-T2)’s $\Delta H_{u,2}$ with crystallinity $x_{c}\approx$ 100% is still unknown, even though Takacs et al. [49] reported remarkable order, “face-on lamella”, in a P(NDI2OD-T2) film. Importantly, Clark et al. [50] estimated the crystallinity ($x_{c}\approx$ 39 ± 10%) of r-reg P3HT (Plextronics and Merck) based on spectroscopic methods. Hence, when we examined r-reg P3HT′s $x_{c}$ based on the previous studies ($\Delta H_{u,r-reg\;P3HT}\approx$47.5 J/g) [2], $x_{c}\approx \left(17.80/47.50\right)\times 100$ = 37.5%, which falls in Clark et al.′s spectroscopic results. In the same vein, Neher et al. [31] estimated that non-amorphous aggregation in P(NDI2OD-T2) (*M*_n_ = 36.2 kg/mol, PDI = 5.0) is about 45% based on their spectroscopic results, following Clark et al.’s approach. Considering our P(NDI2OD-T2)’s *M*_n_ and PDI were 32.1 kg/mol and 2.8, respectively, our P(NDI2OD-T2) was roughly similar to Neher et al.’s polymer. Hence, if we consider that our P(NDI2OD-T2) has an enthalpy of 14.46 J/g with the assumption of $x_{c}\approx$ 45 ± 10%, the $\Delta H_{u,2}$ of P(NDI2OD-T2) with $x_{c}\approx$ 100% is estimated to be ~32.13 ± 7.58 J/g. In Figure 3, LLE is calculated based on Equations (6) and (7), and SLE is based on Equation (9) with $\Delta H_{u,2}\approx 32.13$ J/g. Figure 3a–c shows three representative cases of phase transition in semicrystalline polymer solutions. Note that, if we consider ± 10% error in $x_{c}$, the estimated deviation in the SLE curve is about ± 23 K in average from the SLE curve with $x_{c}\approx 45\%$ for the P(NDI2OD-T2)/CB system [see Figure S1 in Supplementary Materials]. Based on the data shown in Figure 3, three representative cases can be discussed, keeping in mind that the FH lattice theory provides qualitative descriptions. 

**Case 1: LLE > SLE.**

Figure 3a shows the phase behavior of the P(NDI2OD-T2)/CB system, which displays both L–L and L–S phase transition, LLE > SLE when $\phi_{P(NDi2OD-T2)}<0.37$. This kind of phase behavior (LLE > SLE) was also observed in polyethylene (PE)/nitrobenzene, PE/amyl acetate and poly (*N*,*N*′-sebacoylpiperazine)/diphenyl ether systems [51,52]. However, note that r-reg P3HT, PC_61_BM, and PC_71_BM show SLE > LLE in CB [2]. This difference between LLE > SLE and SLE > LLE should make polymer solutions undergo different pathways for morphology formation, in which the former undergo SD or NG, but the latter crystallization. If we use chloronaphthalene (CN, δ = 10.3) [53] or 1,2-dichlorobenzene (DCB, δ = 10.0) [44] or 1,2,4-trichlorobenzene (TCB, δ = 10.2) [53] as a solvent for P(NDI2OD-T2), the phase behavior is included in Case 1, because their solubility parameters are larger than CB′s δ = 9.5, inducing more L–L demixing in solution. The regions in Figure 3a correspond to: (A) one-phase liquid state; (B) two-phase liquid state; (C) both L–L and L–S phase separation; and (D) L–S phase separation (i.e., polymer crystallization), respectively. 

**Case 2: LLE ≈ SLE.**

Figure 3b shows the phase behavior of the P(NDI2OD-T2)/CF system, in which the upper critical solution temperature (UCST), i.e., the binodal coexistence line, is around the melting point depression curve, indicating L–L phase transition may compete with L–S phase transition, i.e., self-assembly for crystallization at $\phi_{P(NDi2OD-T2)}<0.15$. Note that, in Figure 3b, the regions (A), (B), (C), and (D) correspond to each state in Figure 3a except for the minimized (B) region, indicating both L–L and L–S phase transition may occur around the region (B). 

**Case 3: SLE > LLE.**

Figure 3c shows the phase behavior of the P(NDI2OD-T2)/XY system, in which SLE > LLE is displayed. This kind of phase behavior (SLE > LLE) is observed also for r-reg P3HT/CB, PC_61_BM/CB, and PC_71_BM/CB, in which the system undergoes phase separation in solution over crystallization, followed by L–L phase transition. Note that, in Figure 3c, the region (B) completely disappears but the other ones, (A), (C), and (D), exist, only. 

Figure 3d summarizes the phase transition in the P(NDI2OD-T2)/solvent mixtures. Path I generates crystalline aggregation, in which there is equilibrium between a polymeric chain in liquid and self-assembled crystals in aggregation. On the other hand, Path II induces two liquid phases: a polymer-rich phase and a solvent-rich phase. Lastly, as explained in Case 2, it is possible that Paths I and II may compete each other, i.e., simultaneously, the L–L and L–S transitions occur together. 

**Figure 4:**
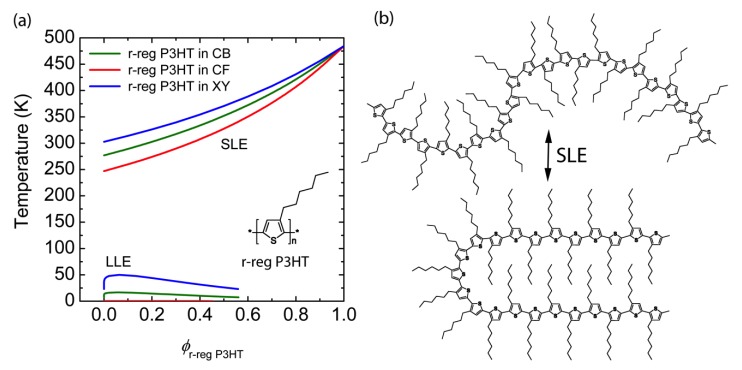
(**a**) Theoretical phase diagrams of binary r-reg P3HT solutions: r-reg P3HT/CB (green solid line), r-reg P3HT/CF (**red**), and r-reg P3HT/XY (**blue**). Inset: Chemical structure of r-reg P3HT. (**b**) Schematic explanation of SLE (L–S phase transition) of r-reg P3HT molecules in solution.

Finally, for the purpose of clear comparison, when we calculated the phase diagrams of r-reg P3HT solution for the same solvent, CB, CF and XY, we can observe only Case 3 (i.e., SLE > LLE) as shown in Figure 4. Hence, we can say that the r-reg P3HT solution may undergo phase separation primarily thorough crystallization, whereas P(NDI2OD-T2) solution may phase separately via SD or NG or crystallization or any combination of SD/NG and crystallization, depending on the solvent, CB or CF or XY. 

### 3.2. Binary Polymer–Polymer Mixture

In the previous section, we noticed that, when CB was used as a solvent, P(NDI2OD-T2) showed LLE > SLE, whereas r-reg P3HT displayed SLE > LLE. In this section, we mixed these two different semicrystalline polymers, r-reg P3HT and P(NDI2OD-T2), in solvents to observe phase behavior. To this end, DSC thermal analysis was employed for some model compositions such as 0, 20, 50, 80, and 100 wt.% P(NDI2OD-T2). Note that two different polymer–polymer systems may be immiscible if there is no specific interaction, because $\Delta G_{M}=\Delta H_{M}-T\Delta S_{M}>0$ $\left(\Delta S_{M}\approx 0\;\mathrm{and}\;\Delta H_{M}>0\right)$, where $\Delta H_{M}\;\mathrm{and}\;\Delta S_{M}$ are the enthalpy and entropy of mixing, respectively. 

**Figure 5:**
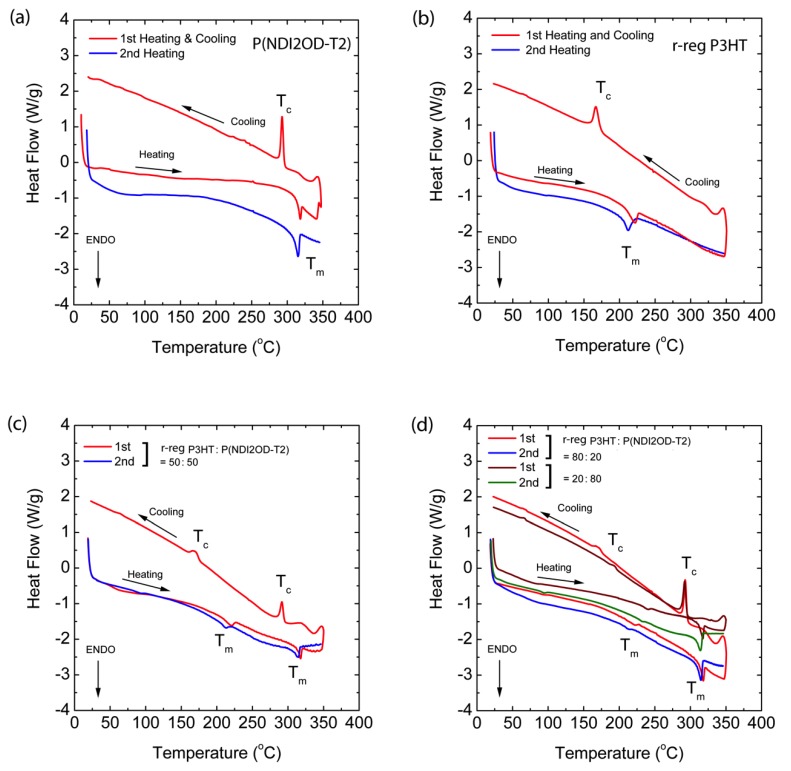
Differential Scanning Calorimetry (DSC) thermograms at a scan rate of 10 °C/min: (**a**) P(NDI2OD-T2), (**b**) r-reg P3HT, (**c**) r-reg P3HT:P(NDI2OD-T2) = 50:50 wt.%, and (**d**) r-reg P3HT:P(NDI2OD-T2) = 80:20 and 20:80 wt.%.

In Figure 5, the red solid lines are the first heating and cooling curves, and the blue line represents the second heating. Through the first heating/cooling cycle, the thermal history of the samples was erased [54] and through the second heating, we may acquire data related to the melting (*T*_m_) and crystallization (*T*_c_) temperatures. The pure polymers, P(NDI2OD-T2) and r-reg P3HT show *T*_m_ at 314.80 °C (with an enthalpy of 14.46 J/g) and at 211.37 °C (that of 17.80 J/g) in Figure 5a,b, respectively. These two polymers were mixed together and compositions of 20, 50, and 80 wt.% P(NDI2OD-T2) were made, resulting in the *T*_m_ and *T*_c_ shown in Figure 5c,d, in which all the blend compositions showed each *T*_m_ and *T*_c_, originating from pure P(NDI2OD-T2) and r-reg P3HT, indicating that these polymers were immiscible, as expected from two different polymer–polymer systems in the absence of any specific interaction. Note that P(NDI2OD-T2) and r-reg P3HT have a similar thermal stability, showing decomposition in the range of ~430–500 °C (Figure S2 in Supplementary Materials). 

**Figure 6:**
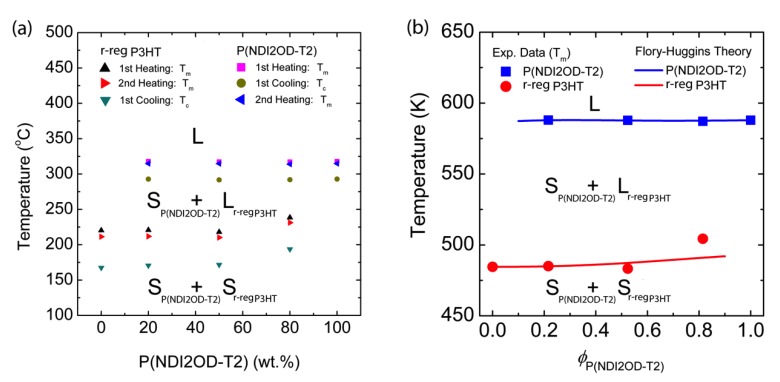
Temperature–composition phase diagram for the binary r-reg P3HT/P(NDI2OD-T2) system, in which **L** and **S** stand for liquid and solid, respectively. (**a**) Experimental results obtained from DSC thermograms in Figure 5 and (**b**) Comparison of experimental data (from the second heating curve) with the Flory–Huggins lattice model (Equations (8) and (10)) with *χ* = 116.8 K/*T* − 0.185.

As a result, based on the information in Figure 5, we constructed the temperature–composition phase diagram of the r-reg P3HT/P(NDI2OD-T2) system (see, Figure 6a), in which the first observation was that *T*_m_ and *T*_c_ were very similar in blends compared to those of each pure polymer, indicating they were immiscible. Note that in the case of the r-reg P3HT/PC_61_BM system, there was a significant melting point depression and miscibility (a miscibility limit at ~40 wt.% PC_61_BM) [55]. Conversely, as shown in Figure 6a, the phase behavior of the r-reg P3HT/P(NDI2OD-T2) system was very simple, in which **L** and **S** stand for liquid and solid state, respectively. When the temperature was lower than *T*_m_ of r-reg P3HT, the resultant phase was two solid mixtures, **S**_P(NDI2OD-T2)_ + **S**_r-reg P3HT_. However, when the temperature was increased above the *T*_m_ of r-reg P3HT but less than the *T*_m_ of P(NDI2OD-T2), the phase was **S**_P(NDI2OD-T2)_ + **L**_r-reg P3HT_. Finally, when the temperature was increased above the *T*_m_ of P(NDI2OD-T2), the phase became a liquid state, **L**. 

Importantly, based on the *T*_m_ data from the second heating curves, we compared the experimental results with the FH model’s prediction (see Figure 6b). For describing the melting points of P(NDI2OD-T2) (i.e., component 2) and r-reg P3HT (i.e., component 1), we used Equation (8) (above) and Equation (10) (below), respectively. Note that the χ interaction parameter for the r-reg P3HT/P(NDI2OD-T2) system was shared together for Equations (8) and (10).

(10) $$\frac{1}{T_{m,1}}-\frac{1}{T_{m,1}^{0}}=-\frac{R}{\Delta H_{u,1}}\frac{V_{u,1}}{r_{1}{\hat{V}}_{1}}\left[\ln\phi_{1}+\phi_{2}\left(1-\frac{r_{2}}{r_{1}}\right)+r_{1}\chi \phi_{2}^{2}\right]$$

where $r_{1}=178\;\mathrm{and}\;r_{2}=193$, and we assume ${\hat{V}}_{1}=V_{u,1}$, indicating the segment volume is decided by the size of r-reg P3HT’s structural unit with the molar volume of 150.9 cm^3^/mol (= 166/1.1) based on the unit molar weight of 166 g/cm^3^ and the density of 1.1 g/cm^3^. Hence, *χ* applies to lattice site volume with the radius of 0.39 nm for r-reg P3HT. Herein, once again the $V_{u,1}/r_{1}{\hat{V}}_{1}\left(=1/r_{1}\right)$ term is related with calculation per r-reg P3HT’s structural unit, because r-reg P3HT is a polymeric chain. And, as in the previous section, when estimating $\chi =\chi_{H}+\chi_{S}$ from the solubility parameter, here also we use $\chi_{H}={\hat{V}}_{1}/RT{\left(\delta_{1}-\delta_{2}\right)}^{2}$. Resultantly, we obtain $\chi_{H}=150.9{\mathrm{cm}}^{3}/\mathrm{mol}/\left(1.987\mathrm{cal}/\mathrm{mol}/KT\right)$ ${\left(9.23-7.99\right)}^{2}\mathrm{cal}/{\mathrm{cm}}^{3}=116.8\;K/T$. However, in this study for the r-reg P3HT/P(NDI2OD-T2) system, if we use 0.34 for $\chi_{S}$, we observe that the theory (Equations (8) and (10)) shows a large deviation from the experimental data (see Figure S3 in Supplementary Materials). Hence, as a first attempt towards describing the data, we employed Equation (11) as:

(11) $$\chi =D\left(T\right)B\left(\phi_{2}\right)=\left(\chi_{H}+\chi_{S}\right)B\left(\phi_{2}\right)=\left(116.8\;K/T+0.34\right)\left(1+b_{1}\phi_{2}+b_{2}\phi_{2}^{2}\right)$$

depending on both temperature and composition vis-á-vis Qian et al.’s extended FH model [48]. However, upon attempting to fit the data using Equation (8) with $\chi =\left(116.8K/T+0.34\right)\left(1+b_{1}\phi_{2}+b_{2}\phi_{2}^{2}\right)$, i.e., in the presence of $\chi_{S}=0.34$ and two adjustable parameters (*b*_1_ and *b*_2_), the theory could not fit the data. 

Alternatively, if we use the entropic part $\chi_{S}$ as an adjustable parameter instead of a fixed value of 0.34, we find that the FH model (Equations (8) and (10)) can describe the experimental data excellently. Resultantly, using Equation (8), we find that, when $\chi_{S}$ is −0.185, the model accurately describes the *T*_m_ of the r-reg P3HT/P(NDI2OD-T2) system. In general, this fitting using an adjustable χ parameter is one of the most common methods in polymer science [54]. Hence, according to Equations (3) and (4) $\left(\chi =\chi_{H}+\chi_{S}=z\Delta w_{H}/kT-z\Delta w_{S}/k;\;\Delta w_{G}=\Delta w_{H}-T\Delta w_{S}\right)$, if $\chi_{S}<0$ and $\Delta w_{S}>0$, this condition indicates an increase of entropy by forming a new contact between the r-reg P3HT and P(NDI2OD-T2) segments [43], although $\Delta S_{M}\approx 0$ in a polymer–polymer mixture. 

Interestingly, Ade et al. reported that poly[N-9′-heptadecanyl-2,7-carbazole-alt-5,5-(4′, 7′-di-2-thienyl-2′,1′, 3′-benzothiadiazole)] (PCDTBT):PC_71_BM mixture has an amorphous–amorphous interaction parameter χ with $\chi_{S}$ = −1.63 or −2.21 (i.e., $\Delta w_{S}>0$) [56], although, in a polymer–solvent system, $\chi_{S}\approx 0.34$ is usually larger than zero (i.e., $\Delta w_{S}<0$), because of the dissimilarity of free volume [43].

To describe in further detail, $\chi_{S}$ is obtained by fitting the *T*_m_ data of P(NDI2OD-T2) in Figure 6b by using Equation (8). Then, using the same value of χ=116.8 K/T−0.185, we described the *T*_m_ of r-reg P3HT in Figure 6b by using Equation (10). As shown in Figure 6, the FH model adequately explains the experimental data. Note that Ade et al. studied the correlation of amorphous–amorphous phase separation and morphologies through the solid-state bilayer inter-diffusion experiments [56], whereas we herein investigated π-conjugated polymer solutions and blends by elucidating a phase-separation mechanism in solutions, affecting the eventual morphologies of a film. 

## 4. Conclusions and Future Work 

In this study, we constructed for the first time phase diagrams of binary n-type low bandgap P(NDI2OD-T2) solutions as a function of three different solvents, chlorobenzene, chloroform, and p-xylene, for which the Flory–Huggins lattice model was employed with a χ interaction parameter estimated from the solubility parameter. The P(NDI2OD-T2) solutions showed the three different types of phase behavior depending on the solvent, Case 1: liquid–liquid equilibria > solid–liquid equilibria, Case 2: liquid–liquid equilibria ≈ solid–liquid equilibria, and Case 3: solid–liquid equilibria > liquid–liquid equilibria, corresponding to P(NDI2OD-T2)/chlorobenzene, P(NDI2OD-T2)/ chloroform, and P(NDI2OD-T2)/p-xylene, respectively. 

Specifically, when we compared the phase behavior of the P(NDI2OD-T2) solutions and that of the r-reg P3HT ones, the latter showed only Case 3: solid–liquid equilibria > liquid–liquid equilibria, indicating r-reg P3HT undergoes phase separation from solutions over crystallization, i.e., liquid–solid phase transition. It is unlikely that r-reg P3HT is phase separated out from solution through spinodal decomposition or nucleation/growth, because the liquid–liquid equilibria are far below the solid–liquid equilibria and room temperature. 

Finally, we constructed the phase diagram of a binary polymer–polymer, the r-reg P3HT/P(NDI2OD-T2) system for the first time. These two semicrystalline polymers showed spontaneous phase separation, i.e., immiscibility, due to both the absence of specific interaction and the entropic penalty coming from the mixing of polymeric chains. Importantly, based on melting point depression theory combined with the Flory–Huggins model, we successfully described the experimental melting points of the r-reg P3HT/P(NDI2OD-T2) system, for which the entropic contribution $\chi_{S}$ to χ was adjusted to be −0.185, indicating an increase of entropy by forming a new contact between two polymer segments. Considering P(NDI2OD-T2) is a commonly used polymer acceptor material for all polymer solar cells, we believe our findings will be of great help for understanding the morphology-generation mechanism for active layers composed of polymer donor and polymer acceptor in all polymer solar cells. 

As a future work, it will be very important to further elucidate the phase-separation mechanism (spinodal decomposition or nucleation/growth or crystallization) experimentally for π-conjugated polymer solutions and blends. 

## Figures and Tables

**Table 1:** Contact angle, surface energy, and solubility parameter.

Materials	Contact Angle (°)	Surface Energy (mJ/m^2^)	Solubility Parameter (cal/cm^3^)^1/2^
P(NDI2OD-T2)	105.40	19.07	7.99
r-reg P3HT	94.90	25.48	9.23

**Table 2:** Solubility parameter [44], molecular weight, molar volume, density, boiling point, and radius of lattice site volume for each solvent, chlorobenzene (CB), chloroform (CF), and p-xylene (XY).

Solvent	Solubility Parameter (cal/cm^3^)^1/2^	Molecular Weight (g/mol)	Molar Volume (cm^3^/mol)	Density (g/cm^3^)	Boiling Point (°C)	Radius of Lattice Site Volume (nm)
CB	9.5	112.56	101.41	1.11	132	0.34
CF	9.2	119.38	80.12	1.49	61	0.31
XY	8.8	106.16	123.44	0.86	138	0.36

**Table 3:** Relative molar volume and Flory–Huggins *χ* interaction parameter for binary P(NDI2OD-T2)/solvent and regioregular (r-reg) P3HT/solvent mixtures as a function of solvent (CB, CF, and XY), when P(NDI2OD-T2) has *M*_n_ = 32.1 kg/mol and δ = 7.99, and r-reg P3HT has *M*_n_ = 29.6 kg/mol and δ = 9.23.

Solvent	P(NDI2OD-T2)/Solvent Mixture		R-reg P3HT/Solvent Mixture	
	$r_{2}$	χ	$r_{2}$	χ
CB	288	116.4 K/*T* + 0.34	265	3.72 K/*T* + 0.34
CF	364	59.0 K/*T* + 0.34	336	0.04 K/*T* + 0.34
XY	236	40.8 K/*T* + 0.34	218	11.49 K/*T* + 0.34

## Supplementary Materials

The following are available online at https://www.mdpi.com/2073-4360/11/9/1474/s1, Figure S1: Theoretical phase diagrams of binary polymer solutions: P(NDI2OD-T2)-CB. Melting point depression curves are calculated for three hypothetical crystallinities of P(NDI2OD-T2), i.e.,$x_{c}$ = 35% (blue line), 45% (red), and 55% (green). Figure S2: TGA thermograms of P(NDI2OD-T2) and r-reg P3HT. Figure S3: Theoretical description of melting points for the binary r-reg P3HT/P(NDI2OD-T2) system by using the theory of melting point depression combined with the Flory–Huggins model incorporating the polymer–polymer interaction parameters of 116.8 K/*T* + 0.340, 116.8 K/*T*, and 116.8 K/*T* – 0.185, respectively.
