# Erratum: Phase Diagrams of *n*-Type Low Bandgap Naphthalenediimide-Bithiophene Copolymer Solutions and Blends. *Polymers* 2019, *11*, 1474

**DOI:** 10.3390/polym11101649

**Published:** 2019-10-11

**Authors:** Gada Muleta Fanta, Pawel Jarka, Urszula Szeluga, Tomasz Tański, Jung Yong Kim

**Affiliations:** 1 Institute of Engineering Materials and Biomaterials, Faculty of Mechanical Engineering, Silesian University of Technology, 44-100 Gliwice, Poland; gadamuleta2019@gmail.com (G.M.F.); Pawel.Jarka@polsl.pl (P.J.); Tomasz.Tanski@polsl.pl (T.T.); 2 School of Materials Science and Engineering, Jimma Institute of Technology, Jimma University, Post Office Box 378 Jimma, Ethiopia; 3 Centre of Polymer and Carbon Materials, Polish Academy of Sciences, M. Curie-Skłodowska 34 Street, 41-819 Zabrze, Poland; uszeluga@cmpw-pan.edu.pl; 4 School of Chemical Engineering, Jimma Institute of Technology, Jimma University, Post Office Box 378 Jimma, Ethiopia

The authors wish to make a change to the published paper [1]. In the affiliation 3: Center of Polymer and Carbon Materials, Polish Academy of Sciences, M. Curie-Skłodowska 34 Street,41-819 Zabrze, Poland. “Center” should be corrected to “Centre”. The authors apologize for any inconvenience caused. The change does not affect the scientific results. The manuscript will be updated and the original will remain online on the article webpage https://www.mdpi.com/2073-4360/11/9/1474.
