# Supplementary material for: Phase Diagrams of n-Type Low Bandgap Naphthalenediimide-Bithiophene Copolymer Solutions and Blends
Source: Polymers (Basel). 2019 Sep 9;11(9):1474. doi: 10.3390/polym11091474 (PMC6780169; doi:10.3390/polym11091474)
Supplement: Supplementary file 1 [file polymers-11-01474-s001.pdf]

Supplementary Materials

Phase Diagrams of n-Type Low Bandgap Naphthalenediimide-Bithiophene Copolymer Solutions and Blends

Gada Muleta Fanta[1,2], Pawel Jarka[1], Urszula Szeluga[3], Tomasz Tański[1], and Jung Yong Kim[2,4,*]

[1]Institute of Engineering Materials and Biomaterials, Faculty of Mechanical Engineering, Silesian University of Technology, 44-100 Gliwice, Poland

[2]School of Materials Science and Engineering, Jimma Institute of Technology, Jimma University, P.O. Box 378, Jimma, Ethiopia

[3]Center of Polymer and Carbon Materials to the Polish Academy of Sciences, M. Curie-Skłodowska 34 Str., 41-819 Zabrze, Poland

[4]School of Chemical Engineering, Jimma Institute of Technology, Jimma University, P.O. Box 378, Jimma, Ethiopia

* To whom correspondence should be addressed:

E-mail: jungyong.kim@ju.edu.et (J.Y. Kim)

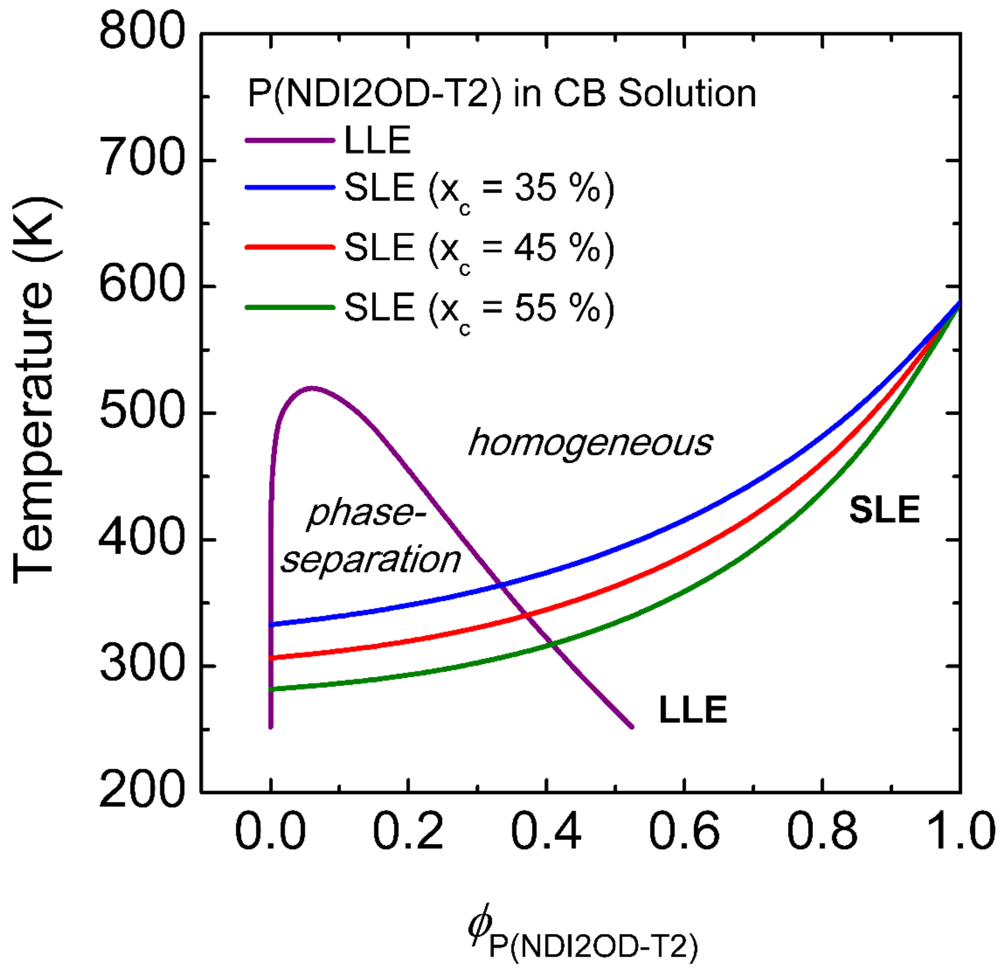

Figure S1. Theoretical phase diagrams of binary polymer solutions: P(NDI2OD-T2)-CB. Melting point depression curves are calculated for three hypothetical crystallinities of P(NDI2OD-T2), i.e., x_c = 35% (blue line), 45% (red), and 55% (green).

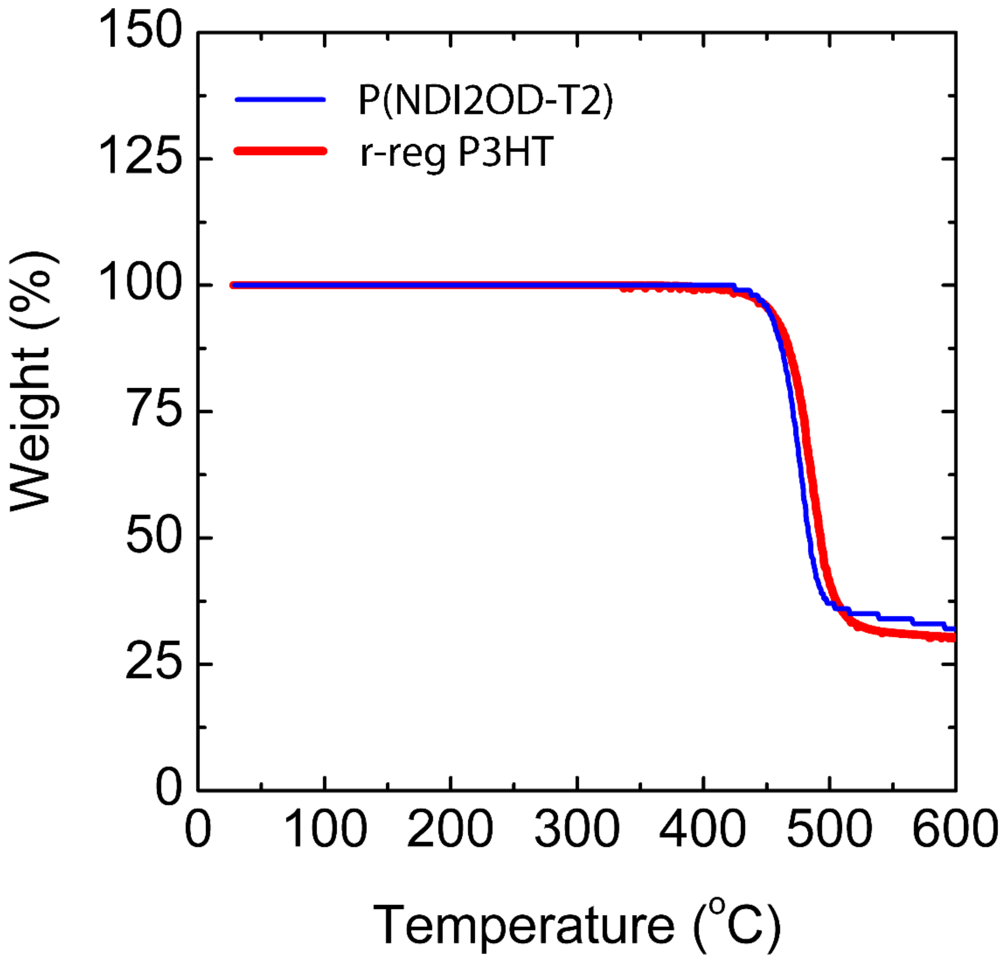

Figure S2. TGA thermograms of P(NDI2OD-T2) and r-reg P3HT.

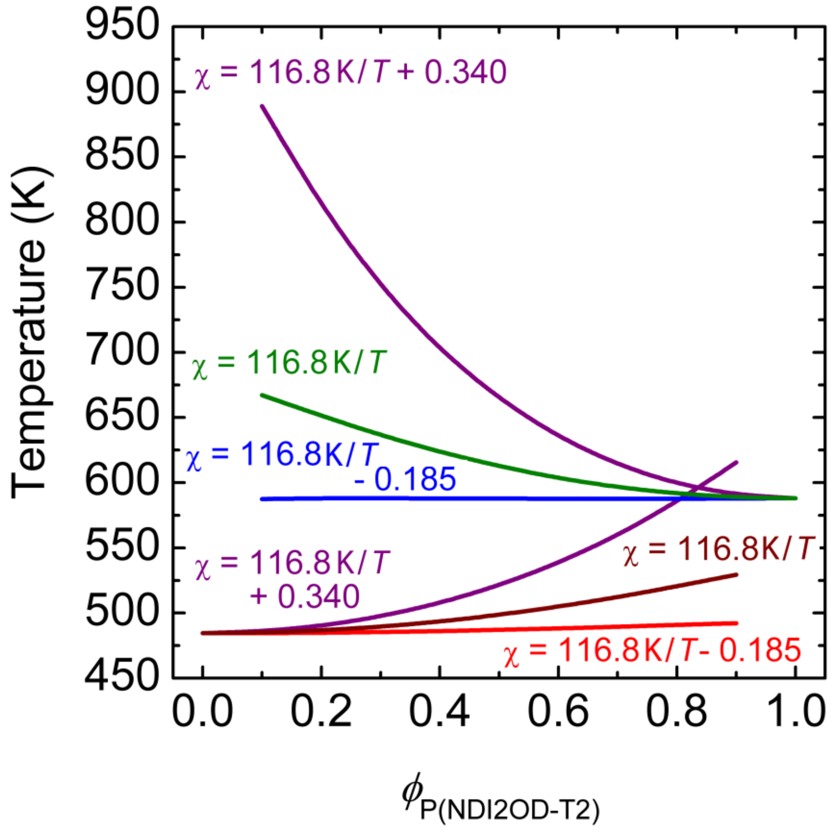

Figure S3.Theoretical description of melting points for the binary r-reg P3HT/P(NDI2OD-T2) system by using the theory of melting point depression combined with Flory-Huggins model incorporating the polymer-polymer interaction parameters of 116.8 K/T + 0.340, 116.8 K/T, and 116.8 K/T – 0.185, respectively.
